# Gut bacteria: an etiological agent in human pathological conditions

**DOI:** 10.3389/fcimb.2024.1291148

**Published:** 2024-10-08

**Authors:** Md Minarul Islam, Nasir Uddin Mahbub, Seong-Tshool Hong, Hea-Jong Chung

**Affiliations:** ^1^ Department of Biomedical Sciences and Institute for Medical Science, Jeonbuk National University Medical School, Jeonju, Republic of Korea; ^2^ Gwangju Center, Korea Basic Science Institute, Gwangju, Republic of Korea

**Keywords:** gut microbiota, probiotic, prebiotic, metabolites, gut-brain axis

## Abstract

Through complex interactions with the host’s immune and physiological systems, gut bacteria play a critical role as etiological agents in a variety of human diseases, having an impact that extends beyond their mere presence and affects the onset, progression, and severity of the disease. Gaining a comprehensive understanding of these microbial interactions is crucial to improving our understanding of disease pathogenesis and creating tailored treatment methods. Correcting microbial imbalances may open new avenues for disease prevention and treatment approaches, according to preliminary data. The gut microbiota exerts an integral part in the pathogenesis of numerous health conditions, including metabolic, neurological, renal, cardiovascular, and gastrointestinal problems as well as COVID-19, according to recent studies. The crucial significance of the microbiome in disease pathogenesis is highlighted by this role, which is comparable to that of hereditary variables. This review investigates the etiological contributions of the gut microbiome to human diseases, its interactions with the host, and the development of prospective therapeutic approaches. To fully harness the benefits of gut microbiome dynamics for improving human health, future research should address existing methodological challenges and deepen our knowledge of microbial interactions.

## 1 Introduction

Trillions of microorganisms, including bacteria, fungi, archaea, protozoa, and viruses, reside within the human body, collectively representing a genetic repertoire that encompasses approximately 100-150 times more genes than the host genome (Thomas et al., 2017; Lkhagva et al., 2021b; Afzaal et al., 2022). Alterations in the microbiome’s composition within an individual can profoundly affect overall health, with modifications in microbial populations potentially leading to considerable changes in health outcomes (Voigt et al., 2015; Gacesa et al., 2022). Their composition is modulated by factors such as mode of birth, feeding system, dietary habits, lifestyle, exercise, drug use, and host genetics (Chang and Kao, 2019; Hasan and Yang, 2019; Lkhagva et al., 2021a; Nandwana et al., 2022; Kok et al., 2023).

The microbiota colonizes the human gut immediately after birth (Milani et al., 2017), though some research has also reported that the microbiome may colonize the human gut during the prenatal period (Perez-Munoz et al., 2017; Xiao and Zhao, 2023). The mode of delivery is a critical determinant in this process. In vaginal deliveries, the infant’s gut is initially colonized by the maternal gut and vaginal microbiota. Conversely, infants delivered via cesarean section are predominantly exposed to skin and environmental microbes from the hospital setting (Dunn et al., 2017). Facultative anaerobes, such as *Escherichia coli*, *Staphylococcus*, and *Streptococcus*, first colonize the infant gut following a vaginal delivery and, for a few days, create an anaerobic environment that allows the survival of strict anaerobes, such as *Bacteroides* and *Bifidobacterium* spp. (Pantoja-Feliciano et al., 2013). Cesarean-section infants are enriched with opportunistic pathogens, including *Enterococcus*, *Enterobacter*, and *Klebsiella* species, and show disturbed transmission of *Bacteroides* and *Bifidobacterium* spp. Breastfeeding can partially restore delayed gut microbiota establishment (Shao et al., 2019; Guo et al., 2020). The mode of early feeding (breastfeeding vs. formula feeding) plays a major role in shaping the gut microbiota during the formative years. Bezirtzoglou et al. (Bezirtzoglou et al., 2011) showed that *Bifidobacterium* species were more than two times higher in breastfeeding infants than in formula-fed infants. Over time, the diversity of the gut microbiota is modulated by a range of factors, including age, sex, dietary intake, pharmacological treatments, physical activity, geographical location, and occupational environment.

The gut microbiome is integral to the maturation of the host immune system, the metabolism of pharmaceuticals, the process of digestion, cognitive function, the neutralization of toxins, the synthesis of essential vitamins, and the establishment of a conducive environment for commensal microorganisms. These functions collectively contribute to pathogen defense and the prevention of severe diseases (Abt and Pamer, 2014; Li et al., 2016a; Mohajeri et al., 2018; Nguyen et al., 2019; Sasso et al., 2023). Probiotic bacteria, often termed “beneficial” or “commensal” microorganisms, are essential for sustaining a balanced and healthful microbial milieu within the digestive system by suppressing pathogenic or “harmful” microbes and regulating the pH of the gut. Dietary fibers and resistant starches, which are not metabolized by endogenous digestive enzymes, are fermented by the gut microbiota to produce short-chain fatty acids (SCFAs) such as acetate, butyrate, and propionate within the colon. SCFAs, particularly butyrate, are vital for maintaining the gut microbiome’s equilibrium through various localized effects, including the preservation of intestinal barrier integrity, enhancement of mucus production, attenuation of inflammation, modulation of immune responses, and potential reduction of the detrimental impacts associated with pathogenic microorganisms (Vinolo et al., 2011; Corrêa-Oliveira et al., 2016; Fang et al., 2021; Vinelli et al., 2022). Non-digestible food supplements, called prebiotics, can selectively stimulate the growth of beneficial microbes, such as *Bifidobacterium* and *Lactobacillus* species (Rastall and Gibson, 2015). Prebiotics contribute to the maintenance of a balanced microbial ecosystem in the gut by supplying essential nutrients to beneficial microorganisms. This support enhances digestive function, optimizes nutrient absorption, and bolsters immune function, while also mitigating the risk of gastrointestinal disorders (Guarino et al., 2020; You et al., 2022).

The disruption of the gut microbiota ecosystem, referred to as dysbiosis, alters the normal gut flora and precipitates the onset of diverse pathologies. This disruption leads to an overgrowth of pathogenic bacteria and a decrease in beneficial microbes, which compromises gut barrier integrity and triggers pro-inflammatory responses and dysregulated immune function. Consequently, dysbiosis is implicated in a wide array of diseases, including neurodegenerative disorders, cardiovascular diseases, metabolic syndromes, gastrointestinal disorders, COVID-19, and colorectal carcinoma, as confirmed by both human and animal studies (Degruttola et al., 2016; Hou et al., 2022). In this review, we encapsulate the most recent research on the impact of the gut bacteria on human diseases, focusing on its underlying mechanisms and regulatory aspects. Moreover, we explicate the potential mechanisms of microbial metabolism and its derivatives that facilitate disease development and progression, offering insights into potential targets for future preventive and therapeutic strategies.

## 2 The impact of the gut bacteria on human health maintenance

Gut microbiota profoundly affects human health by impacting immune system activity, metabolic functions, and the balance of microbial communities essential for physiological stability. The gut microbiota modulates the host’s innate and adaptive immune responses through their components and metabolic products. Metabolites produced by the gut microbiota are crucial in maintaining the fundamental functions of the host. Conversely, disruption in the production of these metabolites can contribute to the pathogenesis of various diseases. Short-chain fatty acids (SCFAs) are microbial metabolites that facilitate the differentiation of T cells into effector T cells and regulatory T cells (Park et al., 2015). The peptides B7 and B12, secreted by *Bifidobacterium longum* and *Bacteroides fragilis*, respectively, play a role in modulating intestinal cytokine production in patients suffering from inflammatory bowel disease (IBD) (Fernández-Tomé et al., 2019). In an unhealthy infant, the gut fosters a pronounced TH1 profile and a pro-inflammatory immune response, leading to the secretion of IL-12 and interferon (IFN)-gamma. This, in turn, causes tissue damage and disrupts normal immune functions (Tamburini et al., 2016). Moreover, the gut microbiome is crucial for the digestion of dietary fiber, as human digestive enzymes are incapable of fermenting non-starch polysaccharides (Holscher, 2017). The short-chain fatty acid butyrate, generated from the microbial fermentation of dietary fiber, is essential for epithelial cell metabolism and functionality. It also promotes the wound-healing response and fortifies epithelial barrier functions in patients with inflammatory bowel disease (IBD) (Wang et al., 2020). Furthermore, it contributes to the regulation of anti-inflammatory responses and shows promise for therapeutic applications in diseases such as intestinal neoplasia and inflammatory bowel disease (IBD) (Salvi and Cowles, 2021). Bifidobacterial strains impede the infection caused by Shiga toxin-producing *E. coli O157:H7*, leading to increased acetate synthesis from carbohydrates (Fukuda et al., 2011). A study involving germ-free (GF) rats demonstrated impaired intestinal transit and diminished small intestinal contractions compared to conventional rats (Husebye et al., 2001). The enteric glial cell network is pivotal in regulating the function of the intestinal epithelial barrier and modulating the intestinal immune response (Inlender et al., 2021). The gut microbiota plays a critical role in the postnatal development of enteric glial cell networks within the intestinal mucosa and influences the colonization of glial cells in the lamina propria of adult mice (Kabouridis et al., 2015). Gut bacteria such as *Bifidobacterium dentium* and *Akkermansia muciniphila* contribute to a healthier gut environment by promoting intestinal mucus production and enhancing goblet cell function, without causing significant degradation of mucin (Engevik et al., 2019; Kim et al., 2021). Additionally, the human gut microbiome participates in the chemical transformation of industrial chemicals and pharmaceuticals into metabolites, which can influence their bioavailability, bioactivity, and toxicity (Koppel et al., 2017). Chemotherapeutic agents, such as doxorubicin, may induce severe gastrointestinal complications, including enteric mucositis and dysbiosis, and the intestinal microbiome has the capacity to modulate these adverse effects (Blaustein et al., 2021). Specifically, the intestinal bacterium *Raoultella planticola* can detoxify the anticancer drug doxorubicin under anaerobic conditions through glycosylation, thereby enhancing host survival. Similarly, *E. coli* and *Klebsiella pneumoniae* strains facilitate the same biotransformation process via analogous biosynthetic pathways, reducing toxicity in the model eukaryote *Caenorhabditis elegans* by generating metabolites (Yan et al., 2018). Furthermore, the intestinal bacterium *Eggerthella lenta* can deactivate the cardiac drug digoxin, converting it to dihydrodigoxin (Haiser et al., 2013). These findings underscore the critical role of the gut microbiome in modulating drug metabolism and its potential implications for therapeutic efficacy and safety. The gut bacteria play a pivotal role in the maintenance of human health by influencing a range of physiological processes, including immune system regulation, nutrient metabolism, and disease prevention. Their intricate interactions with the host contribute to overall well-being and highlight the importance of maintaining a balanced microbiome for optimal health outcomes.

## 3 The role of gut bacteria in disease pathogenesis

**Figure 1:**
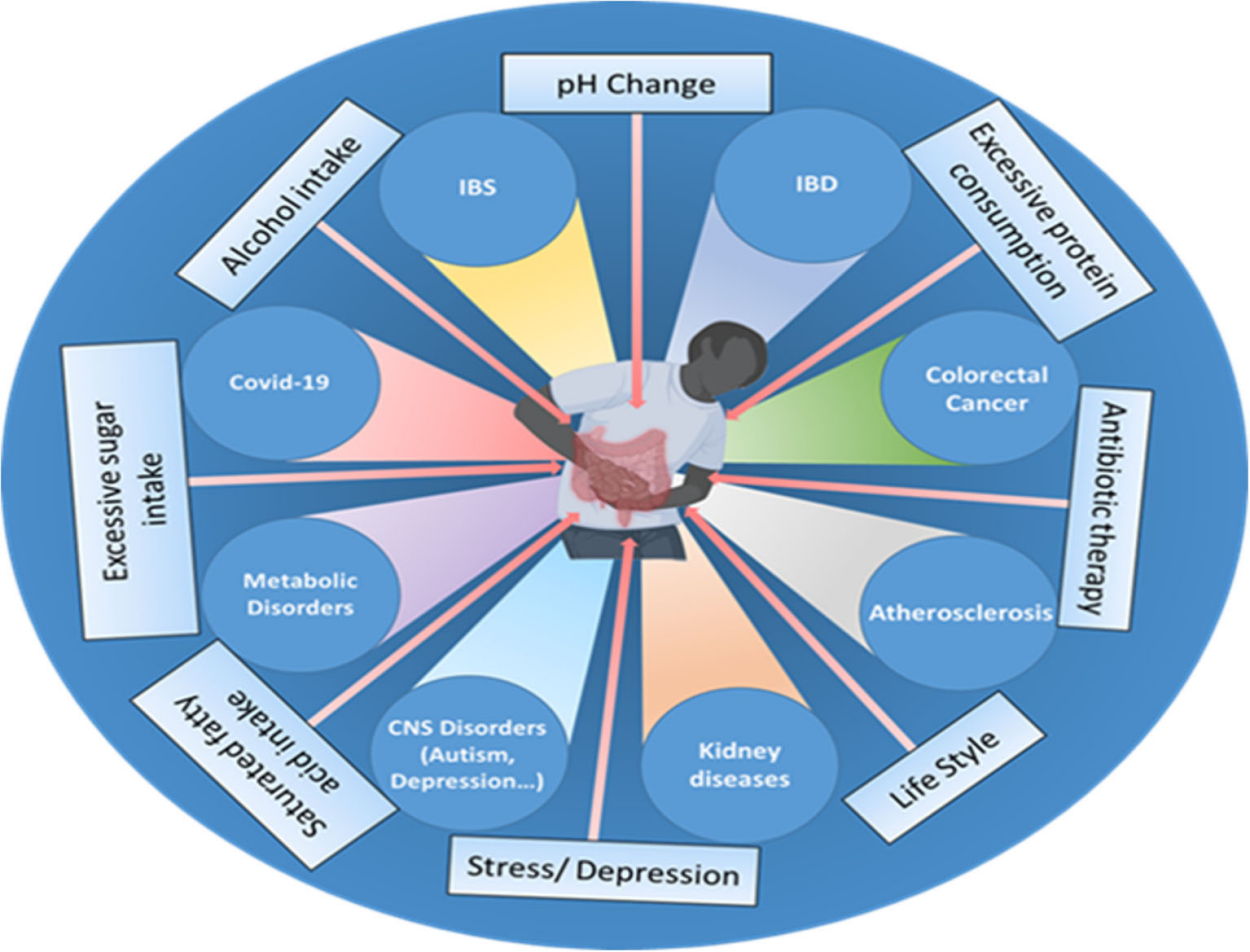
Various factors, such as the use of antibiotics, excessive fat, sugar, and alcohol intake, changes in pH, excessive protein consumption, lifestyle choices, stress, and depression influence the gut microbiome composition and can disrupt the delicate balance of the gut microbiota, leading to various disorders and health conditions such as inflammatory bowel disease (IBD), irritable bowel syndrome (IBS), cardiovascular disease (CVD), colorectal cancer, impaired brain functions, chronic kidney disease (CKD), metabolic dysfunctions and even viral infections like COVID-19.

The human gut bacteria are essential to multiple facets of host physiology and health, with their dysregulation being strongly associated with numerous diseases. The gut microbiome significantly affects gastrointestinal conditions such as inflammatory bowel disease (IBD), irritable bowel syndrome (IBS), chronic constipation, and colorectal cancer (CRC), as well as influencing a spectrum of systemic disorders, including metabolic and neurodegenerative diseases (**Figure 1**).

### 3.1 Gastrointestinal disorders and complications associated with gut bacteria

#### 3.1.1 Inflammatory bowel disease

**Figure 2:**
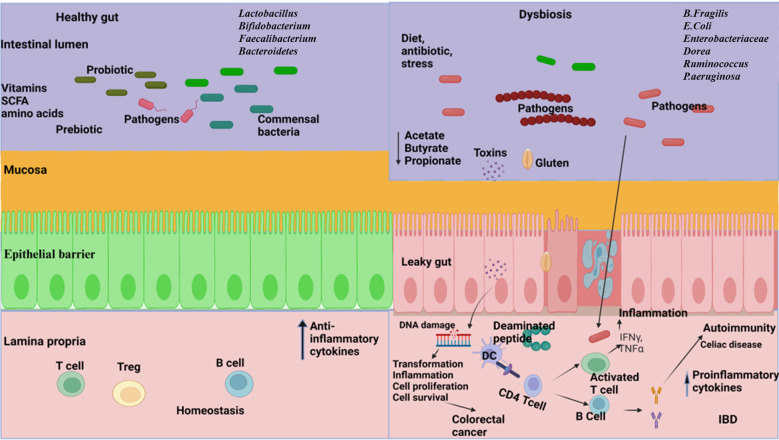
The relationship between gut microbiome and gastrointestinal disorders. Left: A well-balanced gut microbiome promotes normal digestion, optimal nutrient absorption, and supports a strong immune system, effectively protecting against disorders. Right: Dysbiosis in the gut resulting in reduced diversity, abundance of pathogens, increased gut barrier permeability, reduce the production of short chain fatty acids (SCFA), disrupt metabolic processes and trigger dysregulated immune responses leading to the development of inflammatory bowel disease (IBD), celiac disease, and colorectal cancer. (Created with Biorender.com).

Inflammatory bowel disease (IBD), a chronic inflammation of the GI tract, is an umbrella term used to incorporate ulcerative colitis and Crohn’s disease (CD) (Thompson-Chagoyan et al., 2005). The development of IBD is complex and poorly understood due to its multifactorial nature, which involves genetic factors, immune dysfunction, imbalances in gut microbiota, environmental influences, and various aspects of intestinal health (Yuan et al., 2023) (**Figure 2**). The intestinal microbiota plays a crucial role in IBD, as it involves a compromised immune tolerance that results in an excessive immune reaction to gut bacteria. IBD patients frequently exhibit a modified composition of the gut microbiome (Zuo and Ng, 2018; Zhang et al., 2022). Patients with IBD often show a decrease in gut microbiota diversity, particularly in Firmicutes. This reduction can have adverse effects on mucosal integrity and butyrate production, potentially leading to inflammation and impacting cytokine production in the colon (Fernandes and Andreyev, 2022). In patients with CD, anaerobic gram-positive coccoid rods and gram-negative rods are more prevalent than in their healthy counterparts (Van De Merwe et al., 1988); *Bacteroides* and *Bifidobacteria* are poorly associated, whereas *Enterobacteriaceae* are richest in CD (Seksik et al., 2003). The abundance of beneficial bacteria is significantly decreased in patients with IBD. Kang et al. (Kang et al., 2010) found that anti-inflammatory bacteria *Faecalibacterium prausnitzii* and beneficial *Ruminococcus* species are 5-10 times less abundant in patients with inflammatory bowel disease (IBD) compared to the control group. Additionally, pathobionts, which are typically commensal microbes that can become harmful under certain conditions, are often found in higher numbers in individuals with inflammatory bowel disease (IBD) (Nagao-Kitamoto and Kamada, 2017). Gammaproteobacteria become the most abundant when Firmicutes are reduced, and evidence indicates that pathogenic *E. coli* strains accumulate in the intestinal mucosa of patients with IBD, especially in CD (Kang et al., 2010; Lapaquette et al., 2010; Mirsepasi-Lauridsen et al., 2019). Enterotoxigenic *Bacteroides fragilis* colonizes patients with IBD, where *B. fragilis* toxin (BFT) cleaves E-cadherin, reduces mucosal barrier function, and increases epithelial cell proliferation and pro-inflammatory cytokine IL-8 production (Sears, 2009). A recent study conducted on a population revealed a significant link between the use of antibiotics and the likelihood of developing inflammatory bowel disease (IBD), which includes Crohn’s disease and ulcerative colitis. This increased risk was observed across various age groups, especially in cases where the cumulative duration of antibiotic use exceeded 30 days (Aniwan et al., 2018). *Clostridium leptum* group bacteria, including *Faecalibacterium prausnitzii*, were significantly reduced in IBD, shortening the production of SCFAs (Kabeerdoss et al., 2013; Lopez-Siles et al., 2016). Fecal microbiome transplantation might be a potential strategy to fight against IBD. A combination therapy of fecal microbiome with an anti-inflammatory diet helped patients with mild to moderate ulcerative colitis feel better and improved their digestive tracts (Kedia et al., 2022). A pilot study investigated the efficacy of multi-session fecal microbiota transplantation (FMT) in managing active ulcerative colitis (UC). Participants were administered 200 mL of FMT from healthy donors through either colonoscopy or gastroscopy, with evaluations conducted at baseline, week 7, and six months after the intervention. Clinical improvements, including reductions in inflammatory markers, were observed, alongside significant alterations in gut microbiota composition. These findings suggest that multi-session FMT effectively reconstitutes gut microbiota and induces remission in UC patients (Mańkowska-Wierzbicka et al., 2020). Probiotic bacteria, especially engineered probiotics such as *E. coli, Lactobacillus paracasei, Bifidobacterium longum, Lactococcus lactis*, and *Bacteroides ovatus*, are effective therapeutic strategies in the treatment of IBD (Pesce et al., 2022). The administration of intracolonic synbiotic treatment, consisting of *Bifidobacterium animalis subsp. lactis* and xyloglucan, demonstrated significant improvements in mucosal healing and alleviation of colonic symptoms among patients with severe ulcerative colitis (Bozkurt and Kara, 2020).

#### 3.1.2 Irritable bowel syndrome

Irritable bowel syndrome (IBS) is a group of symptoms characterized by abdominal discomfort and irregular bowel movements (Saha, 2014). The pathophysiology of IBS is multifactorial (Surdea-Blaga et al., 2012), including disturbed gut motility (Shaidullov et al., 2021), visceral sensitivity (Li et al., 2020a), neural dysfunction of the gut–brain axis (Suganya and Koo, 2020), autonomous nervous system dysfunction (Mazur et al., 2012), and psychological factors (Van Tilburg et al., 2013), which are implicated in disease progression. Fecal SCFAs are biomarkers for diagnosing IBS (Farup et al., 2016) and are produced by the microbial fermentation of ingestible polysaccharides and proteins, through which commensal microbiota communicate with the host (Rasmussen et al., 1988; Natarajan and Pluznick, 2014). A study of twenty-five subjects with IBS and 25 controls aimed to diagnose IBS by measurement of fecal SCFA; the propionic acid–butyric acid ratio (mmol/l) showed the best diagnostic properties, with a sensitivity of 92% and a specificity of 72% at a cut-off value >0.015 mmol/L (Farup et al., 2016). In a recent study, 490 individuals with IBS and 122 individuals without IBS were analyzed using metagenomic sequencing. In IBS, alpha diversity was significantly lower, rich in gram-negative bacteria, including *Shigella* species, and contained disrupted metabolic pathways associated with short-chain fatty acid and vitamin synthesis, whereas *Eubacterium rectale* and *Faecalibacterium prausnitzii* were relatively sparse in individuals with IBS (Phan et al., 2021). A systematic review and meta-analysis aimed to evaluate the alterations in the intestinal microbiota in IBS using qPCR to quantify bacterial groups. Additionally, there was a significant decrease in *Lactobacillus, Bifidobacterium* and *Faecalibacterium prausnitzii* in IBS compared with the corresponding populations in healthy controls (Liu et al., 2017). Another study reported an increased abundance of *Dorea, Ruminococcus*, and *Clostridium* spp. and a reduction in *Bacteroides, Bifidobacterium*, and *Faecalibacterium* in patients with IBS (Rajilic-Stojanovic et al., 2011). Probiotics and prebiotics are commonly used to treat the symptoms of irritable bowel syndrome (IBS). In a clinical trial, two probiotic strains, *Lactobacillus acidophilus DDS-1* and *Bifidobacterium lactis UABla-12*, were found to improve abdominal pain in individuals with IBS. These probiotics also help regulate bowel movements, potentially relieving diarrhea or constipation, which are common symptoms of IBS (Martoni et al., 2020). Another study showed that supplementation of *Bacillus coagulans MTCC 5856*, along with standard care, was safe and effective in treating diarrhea-predominant IBS in patients, suggesting it as a potential treatment option (Majeed et al., 2016). Furthermore, several other probiotics, including *Lactobacillus bulgaricus, Lactobacillus paracasei, Lactobacillus reuteri, Lactobacillus plantarum, Pediococcus acidilactici*, *Streptococcus thermophilus, Bifidobacterium infantis, Bifidobacterium bifidum, Lactobacillus brevis, Bifidobacterium longum*, and *Saccharomyces boulardii*, have shown positive effects on IBS symptoms (Chlebicz-Wojcik and Slizewska, 2021). The prebiotic galactooligosaccharide promotes the growth of beneficial gut *Bifidobacterium* in IBS patients, leading to a reduction in symptoms such as flatulence, abdominal pain, and discomfort (Silk et al., 2009). Psyllium fiber has also been found to reduce abdominal pain and inflammation in IBS patients (Shulman et al., 2017; Garg, 2021).

#### 3.1.3 Celiac disease

Celiac disease is a critical autoimmune pathology that affects the small intestine. Although gluten is the main trigger, the gut microbiome also has a notable effect on the disease’s progression (Marasco et al., 2016) (**Figure 2**). The gut bacteria from the phyla Firmicutes and Actinobacteria, mostly *Lactobacillus, Streptococcus, Staphylococcus, Bifidobacterium*, and *Clostridium*, is involved in gluten metabolism (Caminero et al., 2014). A previous study showed that rod-shaped bacteria were predominantly associated with the intestinal mucosa of children with active and inactive celiac disease, compared with the bacterial flora seen in the controls (Forsberg et al., 2004). In their culture-dependent method, Collado et al. (Collado et al., 2007) reported that the presence of *Bacteroides, Clostridium*, and *Staphylococcus* was significantly higher in fecal samples from patients with coeliac disease than in healthy subjects. The gluten-free diet is a commonly recommended treatment for patients with celiac disease. However, incorporating probiotics and prebiotics into this diet can potentially restore the gut microbiome, leading to improved gluten breakdown in the gut (Olivares et al., 2014; Drabińska et al., 2020). Consequently, this can reduce inflammation, enhance gut health, and decrease the production of cytokines and antibodies that contribute to issues in celiac disease. As a result, patients may experience fewer symptoms and an overall better quality of life (De Sousa Moraes et al., 2014).

#### 3.1.4 Colorectal cancer

The gut microbiota significantly influences the pathogenesis of colorectal cancer (CRC) through its effects on microbial composition, metabolic activity, and interactions with the host (**Figure 2**). Gut microbial dysbiosis has been reported in patients with CRC, where there is a lower abundance of commensal microbiota, especially SCFA-producing bacteria, and a higher prevalence of pro-inflammatory pathogenic microbes (Sanchez-Alcoholado et al., 2020). Colonizing GF mice with the microbiota of patients with CRC and their healthy counterparts, CRC fecal-receiving mice develop epithelial hyperplasia and DNA methylation in the intestine (Sobhani et al., 2019). *Bacteroides fragilis* is involved in CRC pathogenesis with the production of *Bacteroides fragilis* toxins (BFT) and biofilm (Cheng et al., 2020). Research has highlighted that the bft gene, responsible for encoding BFT toxins, and biofilm formation are essential virulence determinants contributing to colorectal cancer (CRC) pathogenicity. The prevalence of BFT toxin-producing and biofilm-forming strains of *Bacteroides fragilis* is significant among patients with colorectal cancer (Jasemi et al., 2020). However, it has been found that cell-free supernatants from *Clostridium butyricum* can inhibit the growth of *B. fragilis*, prevent biofilm production, and potentially serve as a biotherapeutic agent against CRC (Shin et al., 2020). Studies using quantitative PCR and 16s rRNA gene sequencing methods have shown an increased presence of *Fusobacterium* species and a reduction in the Bacteroidetes and Firmicutes phyla in colorectal carcinoma (Kostic et al., 2012). Research indicated that the supplementation of six viable strains from *Lactobacillus* and *Bifidobacterium* strains significantly lowered levels of proinflammatory cytokines, including TNF-α, IL-6, IL-10, IL-12, IL-17A, IL-17C, and IL-22. Furthermore, this approach was beneficial in reducing the incidence of post-surgical complications in individuals with colorectal cancer (CRC) (Zaharuddin et al., 2019). Another research revealed that a prebiotic formulation containing fructooligosaccharide, xylooligosaccharide, polydextrose, and resistant dextrin had profound impacts on immune-related markers both prior to and following surgical procedures in colorectal cancer (CRC) patients and the administration of these prebiotics led to significant modifications in the populations of commensal bacteria and opportunistic pathogens within the patient cohort (Xie et al., 2019).

### 3.2 Metabolic disorders associated with gut microbiota

#### 3.2.1 Obesity

**Figure 3:**
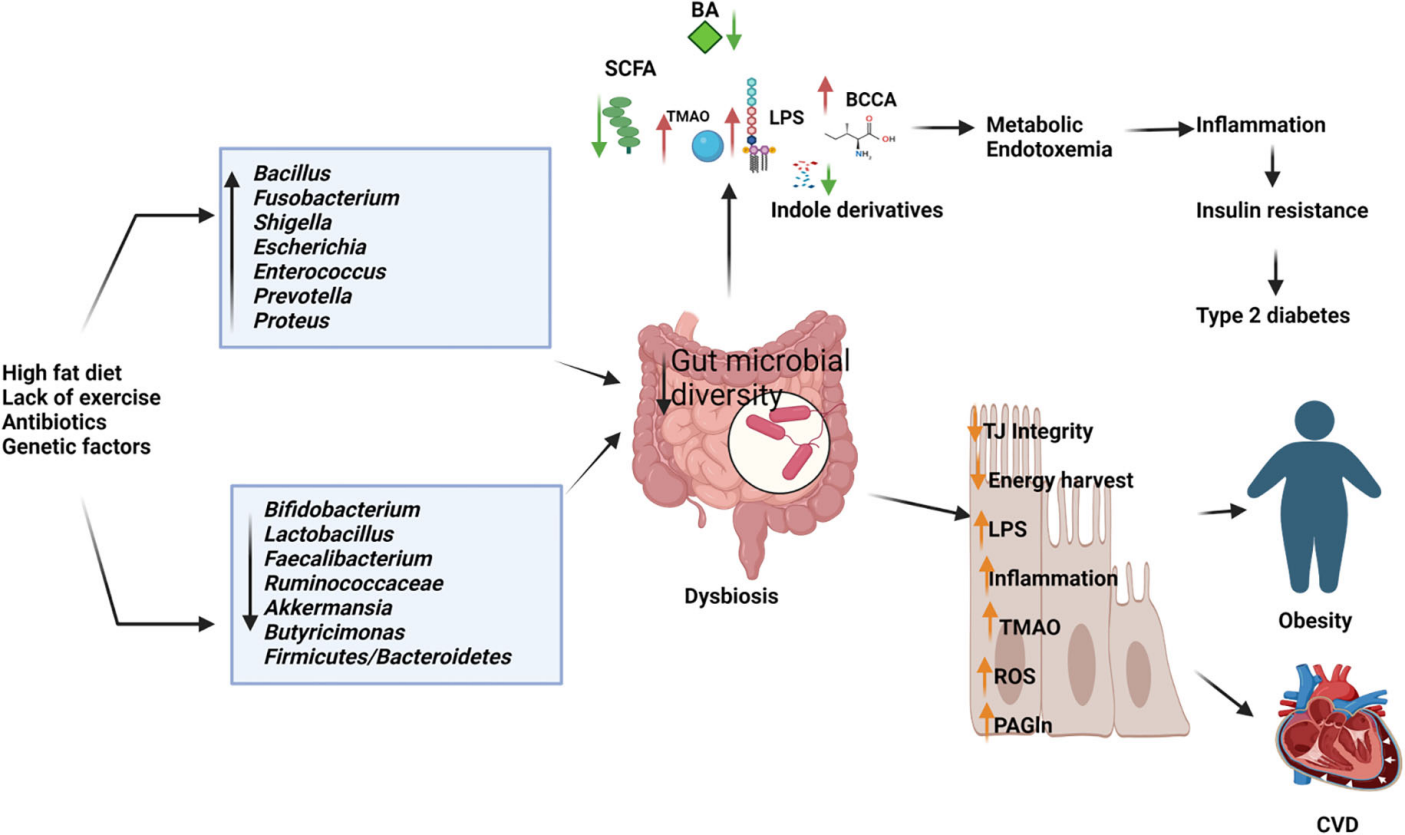
Depletion of beneficial microbiome and abundance of pathogenic microbes in dysbiosis gut causes the elevated production of harmful metabolites and reduces the short chain fatty acids and other beneficial metabolites resulting in type 2 diabetes, obesity cardiovascular diseases (CVD). BA, Bile Acids; LPS, Lipopolysaccharides; BCCA, Branch Chain Amino Acids; TMAO, Trimethylamine N-oxide; ROS, Reactive Oxygen Species; PAGln, Phenylacetylglutamine. (Created with Biorender.com).

Comprehending the intricate interactions between the gut microbiome and obesity is crucial for devising effective strategies to prevent and manage this escalating health concern (**Figure 3**). Emerging evidence suggests an intrinsic link between microbial dysbiosis and obesity (Joseph et al., 2020). Obesity and obesity-associated complications are consequences of alterations in the composition and function of the gut microbiota (Shin and Cho, 2020). Resveratrol intake enhances glucose metabolism and maintains homeostasis, indicating its potential as a therapeutic intervention for obesity. Treating obese mice with resveratrol reduces gut dysbiosis, increases the Bacteroidetes-to-Firmicutes ratio, inhibits the growth of *Enterococcus faecalis*, and promotes the growth of *Lactobacillus* and *Bifidobacterium* (Qiao et al., 2014). Studies with human subjects reported a significant reduction of the Firmicutes/Bacteroidetes ratios in people with obesity compared with those in healthy control (Verdam et al., 2013; Kasai et al., 2015; Duan et al., 2021; Zhai et al., 2022a). In individuals with obesity, beneficial microbiota such as *Bifidobacterium, Faecalibacterium*, and *Ruminococcaceae* are significantly depleted, whereas *Bacillus* and potential opportunistic pathogens such as *Fusobacterium, Escherichia*, and *Shigella* increased (Gao et al., 2018). Mechanistically, the microbiota of individuals with obesity is rich in indigestible polysaccharide-degrading enzymes that produce increased levels of acetate and butyrate (Martinez-Cuesta et al., 2021). Evidence indicates that acetate is linked to obesity, with elevated acetate levels generated by the gut microbiota activating the parasympathetic nervous system, promoting insulin secretion, and leading to hyperphagia and obesity in rodents (Perry et al., 2016). Dietary strategies, such as the administration of probiotics, prebiotics, synbiotics, and fecal microbiota transplants, may facilitate microbial reconstitution and assist in controlling weight gain and associated health conditions. The bacterium *Akkermansia muciniphila* is recognized for its anti-obesity effects. It is well-tolerated and safe for consumption, and has been shown to enhance insulin sensitivity, lower insulin levels, and decrease cholesterol, underscoring its potential role in weight management among individuals with overweight (Depommier et al., 2019). *Lactobacillus plantarum LMT1-48*, for example, can reduce body weight and abdominal fat by regulating lipogenic genes in adipose tissue and the liver (Choi et al., 2020). Oral intake of *Lactobacillus fermentum strain 4B1* has been found to reduce body weight, adipose tissue weight, and adipose cell size, similar to the effects of the drug orlistat (Balolong et al., 2017). *Lactobacillus mali APS1* has been shown to restore the gut microbiome, regulate metabolism and appetite, resulting in weight loss, reduced body fat, liver weight, fat accumulation in the mesenteric adipose depot, and improved hepatic steatosis compared to a diet lacking this probiotic strain (Lin et al., 2016; Chen et al., 2018). Chung et al. (Chung et al., 2016) investigated the efficacy of *Lactobacillus reuteri JBD301* against obesity and found that, like orlistat, *Lactobacillus JBD301* absorbs free fatty acids and excretes them in the feces, leading to significant weight loss in both mice and humans. *Lactobacillus gasseri SBT2055* has multiple benefits for the host, including reduced TAG absorption, accelerated energy expenditure, improved glucose tolerance, increased butyrate production, reduced inflammation, inhibited body weight gain, and decreased fat accumulation. These effects may be linked to the amelioration of adipose tissue inflammation and reduced expression of lipogenic genes in the liver (Miyoshi et al., 2014; Shirouchi et al., 2016). *Bifidobacterium longum BORI* and *Lactobacillus paracasei CH88*, along with fermented ginseng, have demonstrated their ability to reduce various obesity-related markers, including weight gain, lipid deposition, adipocyte size, inflammation, fasting blood glucose levels, and total cholesterol excretion in mouse models and human intervention studies (Kang et al., 2018; Schellekens et al., 2021). Arabinoxylans, a prebiotic derived from rice bran and wheat sources, have the potential to reduce obesity by regulating lipid metabolism, reducing inflammation through the manipulation of gut microbiota, and promoting the production of beneficial short-chain fatty acids. This highlights their potential as prebiotic agents for managing obesity (Neyrinck et al., 2011; Luo et al., 2022).

#### 3.2.2 Diabetes

The gut microbiota also affects diabetes, with disruptions in glucose homeostasis leading to alterations in the host’s gut microbial community, which subsequently contributes to the development of type 2 diabetes mellitus (T2D) and associated comorbidities (Sharma and Tripathi, 2019). An altered gut microbiome deteriorates intestinal barrier function and hosts metabolic and signaling pathways, which results in the progression of T2D (Riedel et al., 2021) (**Figure 3**). Differences in the gut microbiome between patients with T2D and healthy controls have been reported in 16S rDNA amplicon sequencing studies, where butyrate-producing *Faecalibacterium*, *Bifidobacterium*, and *Akkermansia* were significantly reduced in patients with diabetes. The abundance of *Dorea* was significantly increased in individuals with T2D (Li et al., 2020b). A previous study demonstrated that the proportions of the phylum Firmicutes and class Clostridia were significantly reduced in patients with diabetes compared with those in the non-diabetic control group. At the same time, there was an increased presence of Bacteroidetes and Proteobacteria. In addition, the Bacteroidetes to Firmicutes ratio was significantly and positively correlated with reduced glucose tolerance (Larsen et al., 2010). Studies on mice have shown that supplementation with *Bifidobacterium* genus supplements can improve glucose tolerance, insulin secretion, and reduce inflammation (Cani et al., 2007). Metformin and berberine have also been found to be effective in reducing diabetic complications in mice by increasing the presence of SCFA-producing bacteria (such as *Butyricimonas, Coprococcus*, and *Ruminococcus*), while reducing opportunistic pathogens (such as *Prevotella* and *Proteus*), body weight, blood glucose levels, and intestinal inflammation (Zhang et al., 2019). *Lactobacillus* strains, such as *Lactobacillus reuteri 263* and a synbiotic combination of mangiferin and *Lactobacillus reuteri 1-12*, have shown promise in improving insulin resistance, hepatic steatosis, and blood glucose levels in rats fed a high fructose diet (Hsieh et al., 2013; Meng et al., 2023). Other *lactobacillus* strains, including *Lactobacillus rhamnosus BSL, Lactobacillus rhamnosus R23, Lactobacillus plantarum HAC01, Lactobacillus fermentum TKSN041, Lactobacillus gasseri SBT2055, Lactobacillus sakei Probio65, Lactobacillus plantarum Probio-093, Lactobacillus plantarum ZJUFB2, Lactobacillus fermentum MF423, Lactobacillus salivarius AP-32*, and *Lactobacillus reuteri GL-104*, have also shown potential as therapeutic agents for treating type 2 diabetes (Niibo et al., 2019; Farida et al., 2020; Hsieh et al., 2020; Lee et al., 2021; Zhong et al., 2021a; Zhou et al., 2021). In animal models, the probiotic strain *Bifidobacterium animalis 01* has been found to improve glucose metabolism and exhibit hepatoprotective effects, while *Bifidobacterium animalis ssp. lactis GCL2505* (BlaG) reduces symptoms of metabolic syndrome, including visceral fat reduction and improved glucose tolerance, through modulation of the gut microbiota and increased acetate levels (Aoki et al., 2017; Zhang et al., 2020). *Akkermansia muciniphila* has been shown to improve glucose tolerance and insulin sensitivity (Zhang et al., 2021). *Clostridium butyricum* has beneficial effects in reducing high blood sugar levels (hyperglycemia) and promoting the secretion of glucagon-like peptide-1 and insulin (Jia et al., 2017). *Anaerobutyricum soehngenii* has been found to improve insulin sensitivity by stimulating intestinal GLP-1 production (Koopen et al., 2022). Additionally, supplementation with oligofructose-enriched inulin, a prebiotic substance, has been shown to significantly improve glycemic control and reduce inflammatory markers in women with T2D (Dehghan et al., 2014). Emerging therapeutic strategies, including probiotic supplementation, prebiotic intake, and microbial modulation, demonstrate potential in improving glucose metabolism, insulin sensitivity, and reducing diabetic complications. These findings underscore the gut microbiota as a promising target for novel therapeutic approaches in managing and preventing T2D.

### 3.3 The impact of gut bacteria on cardiovascular disorders

Comprehending the interplay between gut dysbiosis and cardiovascular disease offers significant insights into innovative therapeutic and preventive strategies that target cardiovascular risk mitigation through microbiota modulation. Homeostatic processes mediated by host–microbial interactions govern physiological balance and may activate various pathways, thereby contributing to the progression of cardiovascular risk factors (Troseid et al., 2020). Trimethylamine N-oxide (TMAO) and phenylacetylglutamine (PAGln) are metabolites derived from gut microbiota that are linked to cardiovascular disease. Elevated concentrations of TMAO, which is produced by gut bacteria from dietary substrates such as choline, phosphatidylcholine, and L-carnitine, are associated with an increased risk of cardiovascular diseases, including congenital heart disease and atherosclerosis (Chen et al., 2020; Zhen et al., 2023) (**Figure 3**). TMAO induces the production of reactive oxygen species, which induces inflammatory reactions and inhibits the reverse cholesterol transport pathway, resulting in atherosclerosis (Zhu et al., 2020). Phenylacetylglutamine (PAGln) promotes platelet activation and enhances the likelihood of thrombosis by engaging G protein-coupled adrenergic receptors on the platelet surface. This interaction results in receptor activation by PAGln, which causes excessive platelet stimulation, rendering them hyperreactive and contributing to accelerated platelet aggregation and an increased risk of thrombosis (Nemet et al., 2020; Yu et al., 2021). A study suggests the effects of several strains of *Lactobacillus* on atherosclerosis in ApoE-/- mice. They found that this probiotic strain significantly reduced atherosclerotic lesion area, lowered serum lipid levels, and decreased inflammatory markers. The researchers attributed these effects to the probiotic's ability to modulate gut microbiota composition and enhance intestinal barrier function (Zhai et al., 2022b). A study by Li et al. (Li et al., 2016b) explored the effects of *Akkermansia muciniphila* on atherosclerosis in ApoE-/- mice. They found that *A. muciniphila* supplementation reduced atherosclerotic plaque formation, decreased inflammatory markers, and improved gut barrier function. This study highlights the potential of specific probiotic strains in targeting atherosclerosis. Short-chain fatty acids (SCFAs) play a crucial role in regulating anti-inflammatory responses, lipid metabolism, and gluconeogenesis. Bacteria that generate butyrate are instrumental in impeding the advancement of atherosclerosis. Moreover, butyrate demonstrates diverse pharmacological effects, including the promotion of microbial homeostasis, reinforcement of intestinal barrier function, and exertion of anti-inflammatory activities (Amiri et al., 2021). The reduced amount of beneficial or commensal bacteria, including *Faecalibacterium prausnitzii* and *Bacteroides fragilis*, are observed in patients with coronary artery disease (CAD) and type 2 diabetes and increased number of opportunistic pathogens, such as *Enterobacteriaceae, Streptococcus*, and *Desulfovibrio*, have observed in CAD patients without type 2 diabetes. Moreover, patients with CAD-DM2 possessed significantly elevated levels of zonullin and TMAO, the pro-inflammatory cytokine IL-1B, and lower levels of IL-10 (Sanchez-Alcoholado et al., 2017). Gozd-Barszczewska et al. (Gozd-Barszczewska et al., 2017) reported the dominance of Firmicutes and Bacteroidetes in middle-aged men in eastern Poland, with improper levels of total cholesterol and LDL-C rich in *Prevotella* and low in *Clostridium* and *Faecalibacterium*. A Chinese report on patients with atherosclerosis showed a lower abundance of *Bacteroides* and *Prevotella*, which are rich in *Streptococcus* and *Escherichia* (Jie et al., 2017). The opportunistic pathogen *Collinsella* is more prevalent in patients with symptomatic atherosclerosis. In contrast, *Roseburia* and *Eubacterium* are enriched in healthy controls (Karlsson et al., 2012).

Fecal microbiota transplantation (FMT) has demonstrated efficacy in reestablishing microbial equilibrium within the gut and alleviating myocarditis, thereby presenting a potential innovative therapeutic modality for its management (Hu et al., 2019). Modulating the gut microbiota via fecal microbiota transplantation (FMT) has shown a pronounced effect on atherosclerosis in murine models. Transplantation of control microbiota into atherosclerosis-prone mice resulted in a reduction in the advancement of atherosclerotic lesions. In contrast, the transfer of atherosclerosis-prone microbiota into control mice exacerbated lesion progression. These findings indicate that restoring microbial homeostasis in the gut may serve as a viable therapeutic strategy for atherosclerosis (Kim et al., 2022). A study found that clearing gut microbiota with antibiotics and transplanting healthy fecal microbiota could alleviate cardiac fibrosis. This indicates that probiotics, specifically *Clostridium butyricum* and *Bifidobacterium pseudolongum*, as well as metabolite interventions, could offer new strategies for treating cardiovascular disease (Wang et al., 2022). In obese mice with obstructive sleep apnea (OSA) induced by a high-fat high-fructose diet and intermittent hypoxia (IH), both *Lactobacillus rhamnosus* GG (LGG) and LGG cell-free supernatant (LGGs) effectively protected against heart dysfunction, cardiac remodeling, and inflammation. This protection is potentially achieved through the up-regulation of antioxidant pathways mediated by nuclear factor erythroid 2-related factor 2 (Nrf2) (Xu et al., 2019). A six-week regimen of probiotic supplementation in individuals with type 2 diabetes mellitus (T2DM) resulted in significant improvements in cardiovascular disease-related parameters, such as blood pressure and atherogenic indices. Additionally, the Framingham risk score suggested potential benefits in mitigating the risk of future cardiovascular events within this cohort. Nevertheless, further empirical research is necessary to validate these findings (Ahmadian et al., 2022).

### 3.4 Gut bacteria influence on central nervous system pathologies

#### 3.4.1 Anxiety and depression

**Figure 4:**
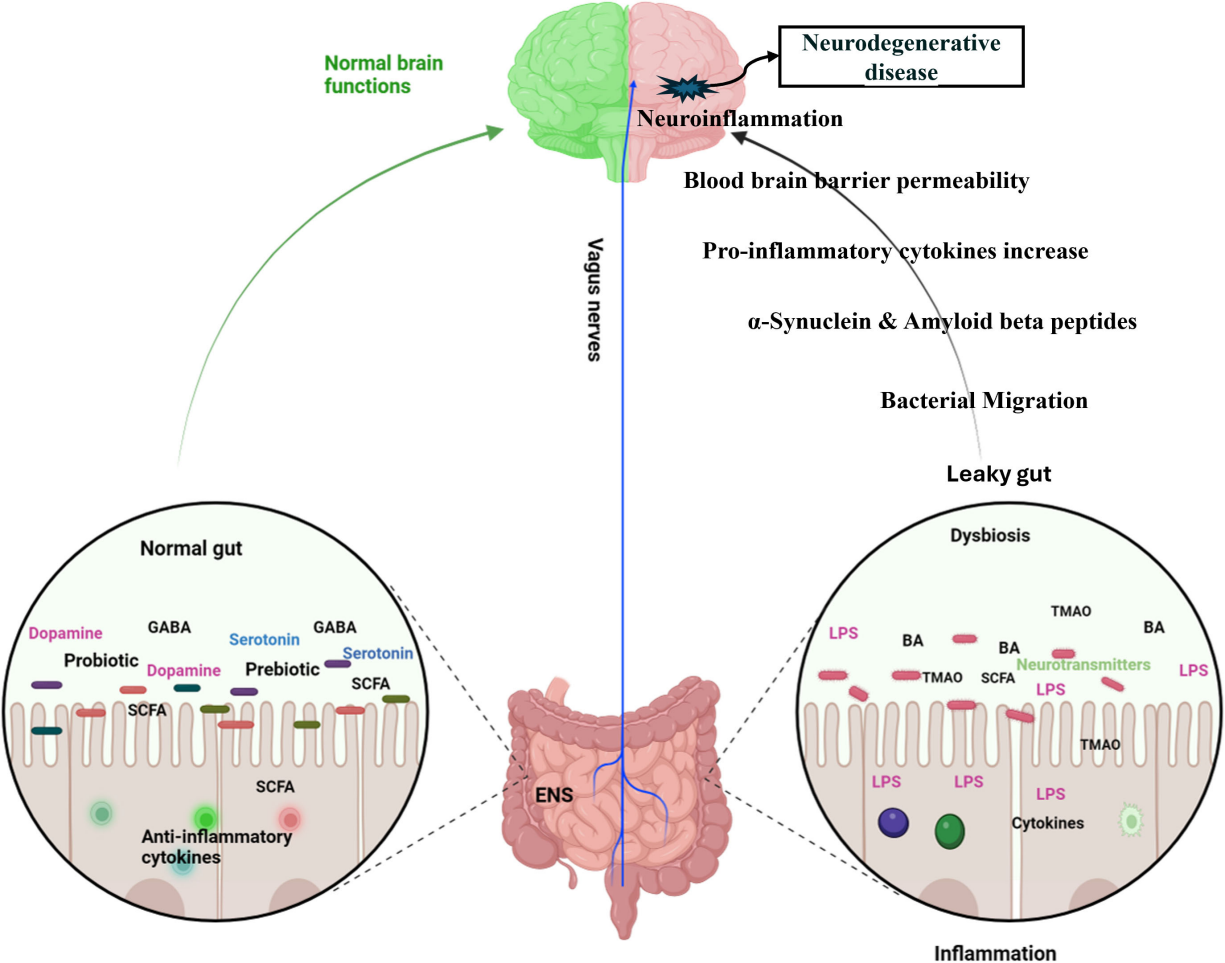
Dynamic communication between the gut, brain, and gut microbiota in both health and disease states. In a state of health (Left diagram), balanced gut microbiome promotes the synthesis of essential metabolites, including short-chain fatty acids (SCFAs), neurotransmitters, and anti-inflammatory cytokines which positively impact brain functions, supporting cognitive health and emotional well-being. In contrast, an imbalance in the gut microbiota (right diagram), disrupts the production of SCFAs, neurotransmitters, and anti-inflammatory cytokines and produces toxic metabolites. These disturbances adversely affect brain health, contributing to cognitive disorders, mood disorders, and inflammation affecting the central nervous system. Specifically, in Alzheimer’s disease, the production of amyloid plaques and neurofibrillary tangles is exacerbated by inflammatory processes and the disruption of gut-brain signaling. In Parkinson’s disease, altered gut microbiota can increase alpha-synuclein aggregation and inflammation, contributing to the degeneration of dopaminergic neurons. GABA, Gamma Amino Butyric Acid; SCFA, Short Chain Fatty acids; ENS, Enteric Nervous System; BA, Bile Acids; LPS, Lipopolysaccharides; TMAO, Trimethylamine N-oxide. (Created with Biorender.com).

The bidirectional relationship between the gastrointestinal (GI) and central nervous systems underscores their close connection. The investigation into the gut microbiota’s role in the gut-brain axis has sparked significant scientific interest, with research focusing on how this axis affects neurodegenerative disorders, including anxiety and depression. Recent studies have explored the potential links between the gut microbiota and these mental health conditions (**Figure 4**). Studies utilizing animal models have indicated that alterations in gut microbiota composition, induced by stress, can influence host behavior and interfere with normal behavioral patterns (Geng et al., 2019; Xu et al., 2020). Compared to conventional specific pathogen-free (SPF) mice, adult germ-free (GF) mice demonstrate reduced anxiety-like behavior in the elevated plus maze, exhibit elevated expression of brain-derived neurotrophic factor (BDNF), and show decreased expression of serotonin (5-hydroxytryptamine) receptors in the brain (Neufeld et al., 2011b; Chen et al., 2013). A subsequent study reported that the offspring of germ-free (GF) mice, which exhibited reduced stress, were colonized with specific pathogen-free (SPF) feces to introduce a normal gut microbiota, followed by a reassessment of anxiety-like behavior. The reconstitution of the gut microbiota did not normalize the behavioral phenotype; anxiolytic behavior persisted in GF mice colonized with SPF microbiota. This indicates that interactions between the gut and the brain are crucial for the development of stress-related systems in the central nervous system (Neufeld et al., 2011a). Increasing evidence highlights the potential of gut bacteria-focused therapies, including fecal microbiota transplantation (FMT), probiotics, prebiotics, and synbiotics, in mitigating anxiety and depression. Studies involving rats have shown that FMT from healthy donors can improve depressive-like symptoms by restoring the gut microbiota, decreasing intestinal inflammation, and reinforcing gut barrier integrity (Rao et al., 2021; Hu et al., 2022). It also progressively alleviated alcohol-induced anxiety and depression in mice (Xu et al., 2018). Fecal microbiome used as adjunctive therapy significantly improved depressive symptoms in patients with depression disorder 4 weeks after transplantation (Doll et al., 2022). In patients with irritable bowel syndrome with diarrhea, fecal microbiota transplantation has been shown to substantially alleviate symptoms of anxiety and depression (Lin et al., 2021a).

Mice fed the probiotic *Lactobacillus rhamnosus* showed decreased depression and anxiety-like behavioral traits (Bravo et al., 2011). *Lactobacillus reuteri NK33* and *Bifidobacterium adolescentis* NK98 alleviate stress, depression, anxiety, and sleep disturbances through the suppression of inflammation, improvement of the gut ecosystem and increased brain-derived neurotrophic factor expression (Jang et al., 2019). Daily supplementation with *Lactobacillus plantarum P8* for 12 weeks notably reduces depression, anxiety, and pro-inflammatory cytokines, while enhancing memory and cognitive traits, particularly in women. This suggests that *Lactobacillus plantarum P8* may be an effective intervention for diminishing these symptoms in stressed adults (Lew et al., 2019).

*Akkermansia muciniphila* and *Clostridium butyricum* were shown to have antidepressant effects in mice with chronic stress by modifying gut microbiota (Ding et al., 2021). They also increased serotonin, brain-derived neurotrophic factor, and glucagon-like peptide-1 (Sun et al., 2018). *Bifidobacterium breve* CCFM1025 and *Bifidobacterium breve* A-1 are two promising psychobiotics. The former reduces depression and related GI disorders by modulating the gut microbiota and tryptophan metabolism, while the latter improves anxiety and depressive symptoms in patients with schizophrenia through altered gut microbiome (Okubo et al., 2019; Tian et al., 2022).

In a randomized controlled trial involving 80 students facing examination stress, a multi-strain probiotic capsule intake with glutamine (*Bacillus coagulans* Unique IS2*, Lactobacillus rhamnosus UBLR58, Bifidobacterium lactis UBBLa70, Lactobacillus plantarum UBLP40* (each of 2x109 CFU); *Bifidobacterium breve UBBr01*, *Bifidobacterium infantis* UBBI01 (each of 1x10^9^ CFU) was found to substantially reduce experienced stress, depression, anxiety, and cortisol levels compared to the placebo group, with no adverse effects (Venkataraman et al., 2021). Another triple-blind, placebo-controlled study investigated the impacts of a multispecies probiotic strain containing *Bifidobacterium bifidum W23, Bifidobacterium lactis W52, Lactobacillus acidophilus W37, Lactobacillus brevis W63, Lactobacillus casei W56, Lactobacillus salivarius W24*, and *Lactococcus lactis W19* and *Lactococcus lactis W58* supplement on cognitive reactivity to sad moods in non-depressed individuals. The study found that the probiotic group experienced a significant reduction in cognitive reactivity to sad mood, particularly in rumination and aggressive thoughts (Steenbergen et al., 2015). Apart from probiotics, prebiotics such as fructo-oligosaccharides and galacto-oligosaccharides have been shown to exert antidepressant effects in mice by altering gut microbiota and enhancing short-chain fatty acid (SCFA) production (Burokas et al., 2017).

#### 3.4.2 Autism spectrum disorder, Parkinson’s disease, schizophrenia, Alzheimer’s disease, and prion disease

Gastrointestinal dysfunction is commonly reported in Parkinson’s disease (PD), schizophrenia [SCZ], autism spectrum disorder (ASD) Alzheimer’s Disease (AD), prion disease, and promotes the onset of these diseases (Pfeiffer, 1998; Davies et al., 2006; Kang et al., 2014; Patrono et al., 2021; Sohrabi et al., 2022; Taniya et al., 2022) (**Figure 4**). Several studies have reported alterations in the gut microbial community in patients with PD/SCZ/ASD compared with the gut microbiota in healthy controls. However, there is little harmony in the results on which groups of microbiomes reduce or increase, and there are even conflicting results (Strati et al., 2017; Zheng et al., 2019; Gorecki et al., 2019; Afroz et al., 2021; Ha et al., 2021; Li et al., 2021b).

In PD *Clostridium* cluster IV, *Akkermansia*, *Bifidobacterium*, and *Lactobacillus* are more abundant, and *Faecalibacterium* spp.*, Coprococcus* spp.*, Blautia* spp.*, Prevotella* spp., and *Prevotellaceae* are significantly reduced in the PD group compared with the corresponding populations in the control group (Gerhardt and Mohajeri, 2018; Bullich et al., 2019). In an alpha-synuclein-overexpressing mouse model, the gut microbiota was required for motor deficits, microglia activation, and alpha-synuclein pathology. Specific microbial metabolites administered orally to GF mice promote neuroinflammation and motor symptoms. Physical impairments were observed in alpha-synuclein-overexpressing mice colonized with microbiota from patients with PD compared with microbiota transplants from healthy human donors (Sampson et al., 2016). Fecal microbiota transplantation (FMT) may exert a positive impact on both motor and non-motor symptoms in individuals with Parkinson’s disease. Over a 12-week treatment period, FMT was associated with increased gut microbiome diversity, reduced constipation, and reported subjective improvements in both motor and non-motor symptoms (Xue et al., 2020; Kuai et al., 2021; Segal et al., 2021; Dupont et al., 2023). In addition, a meta-analysis of nine randomized controlled trials with 663 subjects found that oral probiotic intake substantially improved motor symptoms, gastrointestinal symptoms, anxiety, depression, and reduced laxative use and increased glutathione levels in Parkinson’s disease patients (Chu et al., 2023). Probiotic *Bifidobacterium animalis subsp. lactis* Probio-M8 with conventional drug enhanced sleep patterns, reduced anxiety and GI disorders, and positively changes intestinal microbes and metabolic pathways (Sun et al., 2022a). A mixture of multispecies probiotics containing *Lactobacillus acidophilus, Bifidobacterium bifidum, Lactobacillus reuteri*, and *Lactobacillus fermentum* improved motor behavior, cognitive function, reduced oxidative stress, and neuronal damage in rat model of Parkinson’s disease (Alipour Nosrani et al., 2021).

Aberrant metabolite production by the gut microbiota can modulate immune responses and alter the gut microbiome profile in individuals with autism spectrum disorder (ASD) (Oh and Cheon, 2020). One study reported lower levels of acetic acid and butyrate and an elevated level of valeric acid in patients with ASD. In addition, the butyrate-producing taxa *Ruminococcaceae, Eubacterium, Lachnospiraceae*, and *Erysipelotrichaceae* decreased, and the abundance of valeric acid-associated bacteria (*Acidobacteria*) increased among subjects with autism (Liu et al., 2019). Another study reported that mothers on a long-term high fat diet produced a threat of ASD-like behavior in babies compared with chow-fed mothers, and the gut microbiota differed, with a significant decrease in *Lactobacillus reuteri* observed (Buffington et al., 2016). Fecal microbiota transplantation (FMT) and probiotic supplementation have demonstrated potential in mitigating the disorders associated with autism spectrum disorder (ASD). In a clinical trial, FMT treatment was observed to enhance gastrointestinal (GI) symptoms and behavioral symptoms in children with ASD by modulating gut microbiota composition and altering serum neurotransmitter levels. This treatment reportedly reduced the abundance of *Eubacterium coprostanoligenes* (Li et al., 2021a). Additionally, the probiotic bacterium *Limosilactobacillus reuteri* was found to improve social behavior in a mouse model of neurodegenerative disorders. This improvement was achieved through the vagus nerve, oxytocin, and biopterin pathways which restored synaptic plasticity in the ventral tegmental area (Sgritta et al., 2019; Dooling et al., 2022). A three-month administration of a multispecies probiotic, which includes *Lactobacillus acidophilus, Lactobacillus rhamnosus*, and *Bifidobacteria longum*, has demonstrated the ability to enhance beneficial gut bacteria, decrease body weight, and significantly improve symptoms associated with autism and gastrointestinal disorders (Shaaban et al., 2018). In a case study, a 12-year-old boy with autism spectrum disorder (ASD) underwent treatment using a multi strain probiotic mixture consisting of 10 probiotics, including *Bifidobacterium, Lactobacillus*, and *Streptococcus* genera. The results were astonishing, as the boy experienced significant improvements in his autistic core symptoms after just four months of probiotic treatment (Grossi et al., 2016).

The gut microbiome of individuals with schizophrenia (SCZ) exhibits significant divergence from that of healthy controls or individuals with metabolic syndrome. The SCZ gut is enriched with *Flavonifractor plautii*, *Collinsella aerofaciens, Bilophila wadsworthia*, and *Sellimonas intestinalis*. At the same time, there is a paucity of *Faecalibacterium prausnitzii, Ruminococcus lactaris, Ruminococcus bicirculans*, and *Veillonella rogosae*. Thirion et al. (Thirion et al., 2023) conducted a study to examine the gut microbiome in individuals with schizophrenia (SCZ). The researchers discovered notable differences in the gut bacteria of SCZ patients compared to both the healthy control group and the metabolic syndrome group. Notably, they identified a connection between the biosynthesis of tyrosine by gut bacteria and cognitive function in individuals with SCZ. Antipsychotic agents, including Haloperidol, fluphenazine, chlorpromazine, quetiapine, risperidone, and aripiprazole, are commonly prescribed for the treatment of schizophrenia. However, it is important to note that these medications can have certain side effects related to movement, such as tremors and dystonia. Prolonged administration of these pharmacological agents may result in the onset of uncontrollable orofacial muscle movements (Kang and Kim, 2011; Stroup and Gray, 2018). Conversely, a 12-week intervention involving 60 patients with chronic schizophrenia utilized a probiotic formulation consisting of *Lactobacillus acidophilus, Bifidobacterium bifidum, Lactobacillus reuteri*, and *Lactobacillus fermentum*, along with vitamin D. The findings revealed substantial improvements in both overall and composite Positive and Negative Syndrome Scale (PANSS) scores, signifying a reduction in clinical symptoms (Ghaderi et al., 2019). A randomized, double-blind, placebo-controlled trial was conducted to examine the effects of probiotic supplementation on individuals diagnosed with schizophrenia. The trial utilized a combination of *Lactobacillus rhamnosus strain GG* and *Bifidobacterium animalis subsp. lactis strain Bb12*. The findings indicated that the supplementation did not yield a significant impact on the overall severity of symptoms experienced by these patients. However, it was observed that the supplementation was linked to a decreased likelihood of developing severe bowel difficulties, a prevalent somatic symptom within this population (Dickerson et al., 2014). In addition, complex polyphenols are broken down by the gut microbiota into smaller molecules, which has several health effects including changing the composition of the gut flora. Additionally, polyphenols have important roles in memory, learning, and cognitive processes by shielding neurons from damage and inflammation (Filosa et al., 2018). There is no concrete proof linking the pathophysiology and patho-biochemistry of schizophrenia in humans to a disruption in the intake of polyphenols from food. However, some polyphenols, such as quercetin, have been effectively added to clozapine, an antipsychotic drug used to treat schizophrenia (Schwartz, 2016).Alzheimer’s disease is a progressive neurodegenerative disorder characterized by cognitive decline, memory loss, and behavioral changes. It is marked by the accumulation of amyloid-β plaques and neurofibrillary tangles in the brain (Peddinti et al., 2024). The gut-brain axis involves various pathways, including neural, endocrine, and immune mechanisms, through which the gut microbiota can influence brain function and potentially contribute to the development of AD (Ullah et al., 2023). Gut dysbiosis, an imbalance in the gut microbiota composition, can lead to increased intestinal permeability and the release of pro-inflammatory molecules (Di Vincenzo et al., 2024). These molecules can cross the blood-brain barrier, triggering neuroinflammation and potentially contributing to AD pathogenesis (Peddinti et al., 2024). Some studies have suggested that certain gut bacteria can produce amyloid proteins, which may contribute to the accumulation of amyloid-β in the brain, a hallmark of AD (Chen et al., 2022). Dysbiosis in the gut microbiota can lead to increased oxidative stress, which has been implicated in the pathogenesis of AD (Chen et al., 2022). A study by Li et al. (Li et al., 2019a) found that individuals with mild cognitive impairment and AD had similar alterations in their gut microbiota composition compared to healthy controls. Vogt et al. (Vogt et al., 2017) observed alterations in the gut microbiome of AD patients, suggesting a potential role of gut microbiota in AD pathogenesis. Nagpal et al. (Nagpal et al., 2019) found that a modified Mediterranean ketogenic diet modulated the gut microbiome and short-chain fatty acids in association with Alzheimer’s disease markers in subjects with mild cognitive impairment. This suggests that dietary interventions targeting the gut microbiota may have potential in managing AD. A study by Saji et al. (Saji et al., 2019) revealed a relationship between gut microbiome composition and mild cognitive impairment in patients without dementia, indicating that gut microbiota alterations may precede the development of AD. Saiyasit et al. (Saiyasit et al., 2020). demonstrated that gut dysbiosis develops before metabolic disturbance and cognitive decline in high-fat diet-induced obese conditions. This suggests that gut microbiota alterations may be an early event in the pathogenesis of AD.

Prion diseases, on the other hand, are rare but fatal neurodegenerative disorders caused by the misfolding of prion proteins, leading to brain damage and various neurological symptoms (Soto and Satani, 2011). Some studies have proposed that gut microbiota may influence the misfolding of prion proteins, a key process in the development of prion diseases (Mahbub et al., 2024). Gut microbiota plays a crucial role in maintaining intestinal barrier integrity. Disruption of this barrier could potentially facilitate the entry of prions into the body (Mahbub et al., 2024). The gut microbiota’s influence on the immune system may affect the body’s response to prion proteins and their accumulation (Mahbub et al., 2024). Quigley et al. (Quigley, 2017) reviewed the role of the microbiota-brain-gut axis in neurodegenerative diseases, including prion diseases. The review highlighted the potential influence of gut microbiota on protein misfolding and neuroinflammation, which are key processes in prion diseases. Although not specifically focused on prion diseases, research by Cox and Weiner (2018) on microbiota signaling pathways in neurologic diseases provides a framework for understanding how gut microbiota might influence prion disease progression (Cox and Weiner, 2018). The relationship between gut microbiota and neurodegenerative disorders, particularly Alzheimer’s disease and prion diseases, represents a promising area of research with potential implications for disease prevention and treatment. The gut-brain axis emerges as a critical pathway through which gut microbiota may influence the development and progression of these disorders. This growing body of evidence linking gut microbiota to neurodegenerative disorders opens new avenues for understanding and potentially treating these complex diseases. As our knowledge in this field expands, it may lead to novel therapeutic approaches and preventive strategies, offering hope for those affected by Alzheimer’s disease, prion diseases, and other neurodegenerative disorders.

### 3.5 Mechanistic insight of gut bacteria on renal pathology

**Figure 5:**
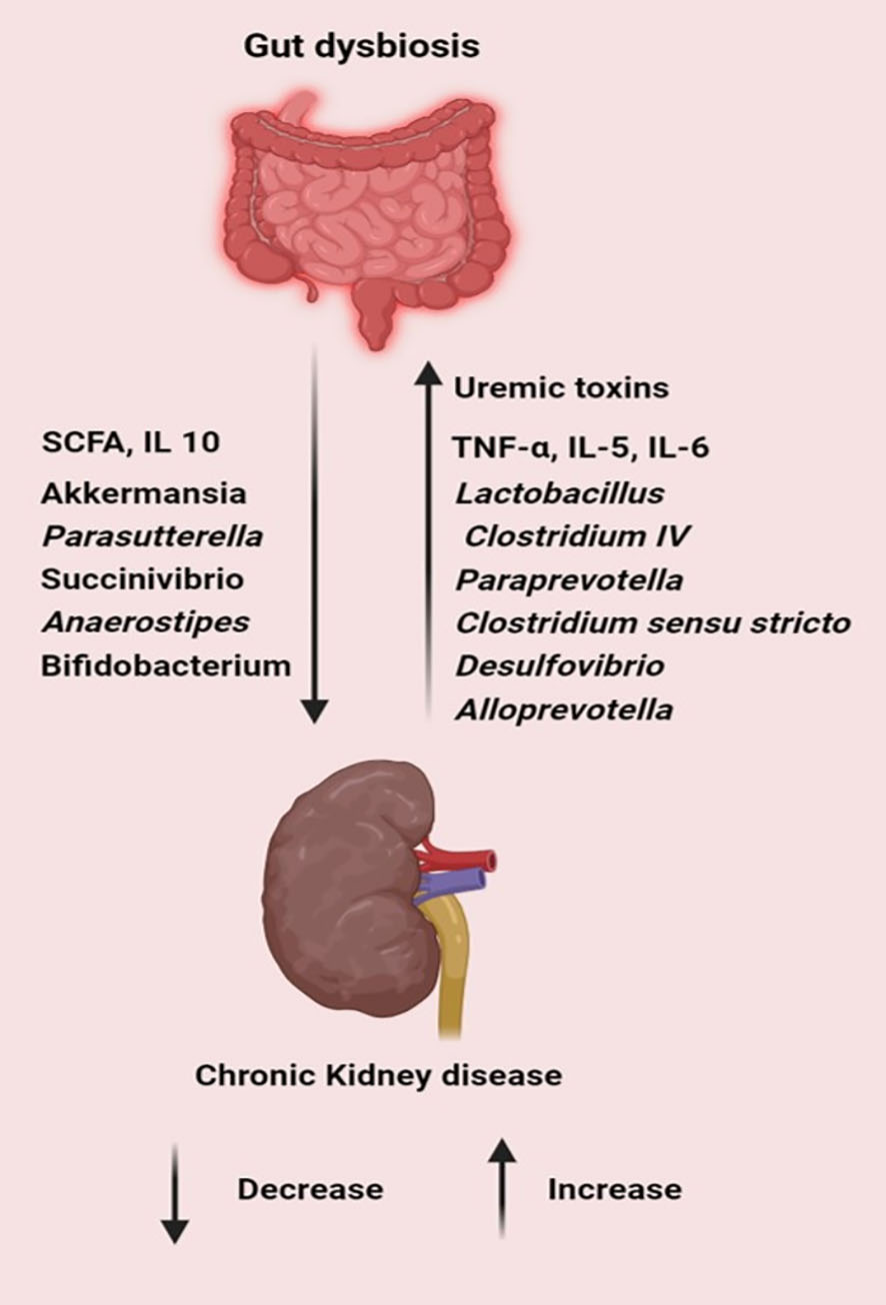
Gut dysbiosis increases pathogens, toxins and inflammatory cytokines while decreasing the level of SCFA, commensal and probiotic bacteria leading to the progression of chronic kidney diseases. (Created with Biorender.com).

Renal disorders, encompassing a range of pathological conditions affecting kidney function, are intricately linked with alterations in the gut microbiota. Recent studies have elucidated several mechanisms by which gut microbiota dysbiosis influences renal health. The microbial fermentation of proteins and amino acids in the gut leads to the generation of excessive toxic metabolites, such as ammonia, amines, thiols, phenols, and indoles, while concurrently diminishing the production of short-chain fatty acids (SCFAs). In the context of chronic kidney disease (CKD), the heightened levels of uremic toxins promote intestinal dysbiosis. This dysbiotic condition undermines the integrity of the intestinal epithelial barrier, thereby aggravating renal damage (Ramezani et al., 2016). A study on patients with CKD showed an increased abundance of *Lactobacillus, Clostridium IV, Paraprevotella*, *Clostridium sensu stricto, Desulfovibrio*, and *Alloprevotella* in the fecal samples of patients with CKD, with a decreased presence of *Akkermansia* and *Parasutterella* compared with the corresponding populations in healthy control subjects (Li et al., 2019b). Low microbial diversity and higher levels of pro-inflammatory cytokines were observed in deceased patients with CKD compared with those in survivors. SCFA-producing bacteria *Succinivibrio* and *Anaerostipes* were considerably lower in non-survivors (Lin et al., 2021b) (**Figure 5**).

With the deepening insight into the microbiome, contemporary research endeavors aim to exploit its potential for enhancing health outcomes in patients with chronic kidney disease (CKD). The most frequently utilized probiotics are *Bifidobacterium* and *Lactobacillus* species. *Bifidobacteria* are recognized for their capacity to ameliorate epithelial damage, restore epithelial functionality, and synthesize short-chain fatty acids. Additionally, they play a pivotal role in the biosynthesis of vitamins and facilitate the proliferation of *Lactobacillus*. Both *Lactobacillus* and *Bifidobacterium* have immune-enhancing and gut mucosal barrier-stabilizing effects (Sanekommu et al., 2023). *Bifobacterium bifidum A218*, *Bifidobacterium catenulatum A302, Bifidobacterium longum A101*, and *Lactobacillus plantarum* combined probiotic administration in peritoneal dialysis patients; serum TNF-α, IL-5, IL-6, and endotoxins significantly decreased, while levels of serum IL-10 significantly increased compared with the corresponding parameters in the placebo group (Shemin et al., 2001). A probiotic mixture consisting of *L. acidophilus KB27, B. longum KB31*, and *S. thermophilus KB19*, when administered orally, has been found to effectively reduce blood urea nitrogen (BUN) levels. This probiotic formulation not only improves the quality of life but is also well-tolerated. These findings support the potential of probiotics in extracting uremic toxins from the intestines (Ranganathan et al., 2010; Vitetta et al., 2019). Elevated levels of Indoxyl sulfate, a uremic toxin, are often associated with kidney dysfunction. Furthermore, a study has shown that oral administration of *Bifidobacterium longum* in a gastro-resistant seamless capsule can effectively reduce serum levels of indoxyl sulfate in hemodialysis patients by rectifying intestinal microflora (Takayama et al., 2003; Taki et al., 2005). *Sporosarcina pasteurii* has emerged as a promising candidate for an “enteric dialysis” application. It has exhibited the capacity to catabolize uremic solutes within the gastrointestinal tract, withstand gastric conditions, and efficiently remove urea *in vitro*. Furthermore, it has been shown to positively influence fermentation processes within the intestinal microbiota. Preliminary findings in nephrectomized rodent models suggest its potential efficacy in alleviating uremic toxicity associated with chronic kidney disease (Ranganathan et al., 2006). Plasma p-cresol, a compound detectable in the bloodstream, is implicated in renal dysfunction and classified as a uremic toxin when present at elevated concentrations. This association poses significant health risks for individuals with chronic kidney disease. A potential therapeutic approach involves synbiotic treatment, which combines probiotics such as *Lactobacillus casei* strain Shirota and *Bifidobacterium breve* strain Yakult with prebiotics like galacto-oligosaccharides. This intervention has demonstrated efficacy in reducing serum p-cresol levels and may mitigate the adverse effects of p-cresol, including its role as a protein-bound uremic toxin contributing to constipation (Nakabayashi et al., 2011). Furthermore, the use of prebiotic gum acacia has shown promising results in altering microbial composition, replenishing depleted butyrate levels, and displaying anti-inflammatory and antioxidant properties. These findings suggest that it could play a beneficial role in the treatment of CKD (Lakshmanan et al., 2021). Additionally, prebiotic D-serine has shown potential as both a therapeutic target and a biomarker for acute kidney injury (AKI), highlighting the complex relationship between gut microbiota and kidney health (Nakade et al., 2018). In essence, probiotics, prebiotics, and synbiotics offer substantial therapeutic benefits for kidney diseases by ameliorating disruptions in gut microbiota and rectifying associated metabolic disorders.

### 3.6 Influence of COVID-19 on gut microbiota composition

**Figure 6:**
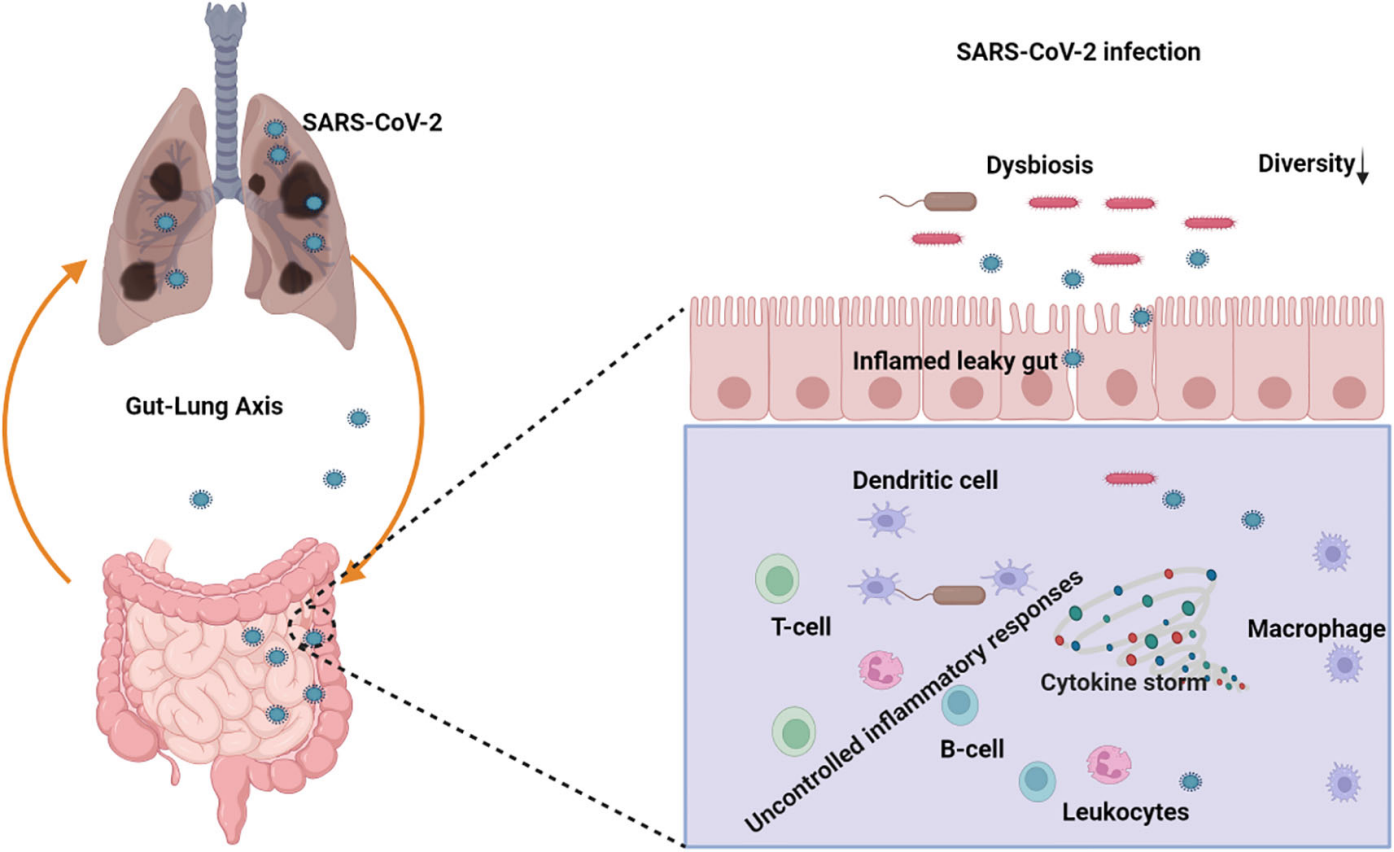
Schematic representation of the role of the gut-lung axis and its potential implications in SARS-CoV-2 infection management with the gut microbiota dysbiosis. (Created with Biorender.com).

Dysbiosis of the gut microbiome can precipitate alterations in immune responses and elevated concentrations of pro-inflammatory cytokines, potentially culminating in a cytokine storm during severe acute respiratory viral infections (**Figure 6**). Emerging studies have revealed disruptions in the gut microbiome among COVID-19 patients, both during and after the disease course. Notable findings include diminished bacterial diversity in older individuals, a decrease in beneficial microbes, an increase in facultative anaerobic bacteria, and a decrease in the production of key metabolites (Bosco and Noti, 2021). This phenomenon may be associated with the higher mortality rate observed in older individuals compared to younger populations in the context of COVID-19 (Casas-Deza et al., 2021). Immunomodulatory bacteria, such as *Faecalibacterium prausnitzii, Eubacterium rectale*, and *bifidobacterial* species, are reduced in patients with COVID-19 (Yeoh et al., 2021). Elevated populations of *Burkholderia contaminans, Bacteroides nordii, Bifidobacterium longum*, and *Blautia* spp. were observed; CAG 257 causes severity in patients with COVID-19 (Sun et al., 2022b). Another study revealed that the *Burkholderia cepacia* complex, *Staphylococcus epidermidis*, or *Mycoplasma* sp. were the most abundant in severely ill patients (Zhong et al., 2021b). Zuo et al. (Zuo et al., 2020) reported that the abundance of *Coprobacillus, Clostridium ramosum*, *and Clostridium hathewayi* was positively correlated with COVID-19 severity. In contrast, there was an inverse correlation between *Faecalibacterium prausnitzii* and disease severity in 15 hospitalized patients.

International efforts have implemented comprehensive COVID-19 vaccination campaigns. However, the emergence of variant strains poses a potential risk to the effectiveness of these vaccines. Additionally, emerging evidence suggests that the gut microbiome may play a critical role in naturally enhancing vaccine responses and modulating immune functions within the intestinal environment (Zimmermann, 2023). A balanced gut microbiota is crucial for combating COVID-19, as it contributes to maintaining a stable immune system that can effectively counteract viral infection. An optimal gut microbiota facilitates the regulation of pro-inflammatory and anti-inflammatory metabolites, thereby preventing excessive inflammation and attenuating the severity of COVID-19 (Chen et al., 2021).

## 4 Concluding observations

The involvement of gut bacteria as significant etiological agents in several human pathological conditions is paramount. As research continues to uncover the intricate relationships between gut microbiota and human health, it becomes increasingly evident that addressing microbial imbalances could offer novel avenues for disease prevention and treatment. Future research endeavors should concentrate on overcoming prevailing methodological constraints and expanding our comprehension of gut microbiome dynamics to optimally utilize its potential for enhancing human health. Despite its importance, progress in elucidating the microbiome’s etiological mechanisms and in creating therapeutic interventions derived from it has been impeded by the limitations inherent in current analytical approaches. Contemporary research on the gut microbiome predominantly employs culture-independent metagenomic next-generation sequencing (mNGS) techniques (Chung et al., 2018). Although mNGS offers valuable insights into the etiological role of the gut microbiome in disease, it is not without constraints (Liu et al., 2022b). This necessitated supplementary culture-dependent experiments (Malla et al., 2018). The essence of such culture-based studies traces back to Koch’s postulates, which necessitate culturing potential etiological agents to establish disease causality (Walker et al., 2006). To fully comprehend the etiological significance of the gut microbiome, pure cultures of the implicated species are indispensable. Such cultures facilitate whole-genome sequencing and enrich metagenome sequencing data resolution (Liu et al., 2022a).

Nevertheless, many gut microbes classified as “unculturable” remain inaccessible to current cultivation techniques, primarily due to the lack of appropriate culture media. Concurrently, the scientific community faces a major challenge in creating a comprehensive gut microbe repository, which is intrinsically linked to the unavailability of a universally applicable culture medium suitable for the diverse microbial entities within the human gut microbiome. Advancing our understanding necessitates prioritizing the development of a complete gut microbe library and the formulation of universal culture media. These limitations are the principal barriers to fully comprehending the etiological roles of the gut microbiome in human diseases. Addressing these challenges is essential for unlocking the full potential of microbiome research and developing effective therapeutic interventions.
